# Synthesis, target analysis, and cerebroprotective effects of novel imide antioxidants via the Nrf2/HO-1 pathway in cerebral ischemia-reperfusion injury

**DOI:** 10.3389/fphar.2025.1552717

**Published:** 2025-05-02

**Authors:** Lili Huang, Yinqi Chen, Hua Zhou, Huihui Chen, Xiping Wu, Zhuochao Wu, Zhiwei Zheng, Zhoudi Liu

**Affiliations:** 1 Lihuili Hospital Affiliated to Ningbo University, Ningbo, China; 2 Shaoxing Second Hospital, Shaoxing, China; 3 Zhejiang Provincial People’s Hospital, Affiliated People’s Hospital, Hangzhou Medical College, Hangzhou, China; 4 Department of Pharmacy, Shaoxing People’s Hospital, Shaoxing, China

**Keywords:** cerebral ischemia-reperfusion injury, oxidative stress, antioxidant, network pharmacology, molecular docking, Nrf2 signaling pathway

## Abstract

**Background:**

Cerebral ischemia-reperfusion injury (CIRI) is a secondary brain injury that occurs after thrombolysis and is a primary cause of death in ischemic stroke patients. Antioxidants that effectively reduce oxidative stress are an efficient treatment approach for CIRI. Here, a novel diimide compound was synthesized using the chemical structure of previously designed anti-inflammatory skeletons.

**Methods and results:**

The antioxidant activities of five compounds (Z1–Z5) were preliminarily evaluated using the hydrogen peroxide-induced PC12 cell damage model, of which Z3 exhibited the best antioxidant effect, even exceeding that of the positive control (tert-butylhydroquinone). Enrichment analysis using network targeting and network pharmacology methods predicted seven candidate core target genes of Z3 in CIRI. Of these targets, computer molecular docking analysis predicted that Z3 has the strongest binding affinity for nuclear factor erythroid 2-related factor (Nrf2). MTT and colony formation assays, reactive oxygen species analysis, immunofluorescence, and immunoblotting experiments verified that Z3 reduced reactive oxygen species to play a protective antioxidant role via the Nrf2/hemoxygenase 1 (HO-1) pathway. The protective effect of Z3 *in vivo* was explored through TTC staining and neurobehavioral scoring of CIRI model mice.

**Conclusion:**

This study provides a new drug development strategy and candidate drug for the treatment of CIRI, offering ideas for the design of new antioxidants.

## 1 Introduction

Cerebral ischemia-reperfusion injury (CIRI) is a secondary brain injury that occurs following thrombolytic therapy in patients with ischemic stroke (Wu et al., 2023). As a severe complication of thrombolytic therapy, CIRI presents a challenge in clinical treatment (Won et al., 2015; Hosoo et al., 2017). Oxidative stress injury has been identified as a critical factor in the complex pathophysiological mechanisms underlying CIRI (Wang et al., 2024; Liu et al., 2015). The excessive production of reactive oxygen species (ROS) during CIRI disrupts normal cellular functions, diminishes enzyme activities, and damages lipids, proteins, DNA, and other macromolecules, which may ultimately induce brain cell apoptosis and tissue necrosis (Liu et al., 2015; Huang et al., 2023). Therefore, the use of antioxidants to eliminate ROS represents a crucial strategy for the prevention and treatment of CIRI (Wang et al., 2024). However, with the exception of a few medications, such as edaravone (ED), few antioxidant agents have been introduced into clinical treatment for CIRI (Kobayashi et al., 2019; Antonic et al., 2018; Schmidt-Pogoda et al., 2020; Wang et al., 2022). Consequently, the identification and development of safe, effective, highly efficient, and low-toxicity novel antioxidants for the treatment of CIRI are urgently needed.

**FIGURE 1:**
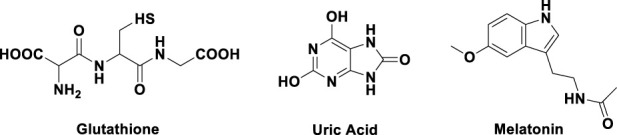
Typical nitrogen-containing antioxidants.

Nitrogen-containing compounds, as exemplified by glutathione, uric acid, and melatonin, constitute one of the primary categories of antioxidants (Saka et al., 2021; Averill-Bates, 2023) (Figure 1). These nitrogen–hydrogen compounds possess lone electron pairs, and can react with ROS to neutralize them. Furthermore, Adamic et al. (Adamic et al., 1969) has demonstrated that such compounds directly interact with ROS through mechanisms involving the physicochemical properties of the nitrogen–hydrogen bonds. Building on these findings and our previous research, our team designed and synthesized a series of novel nitrogen-containing antioxidant compounds featuring a diimide functional group (Z1–Z5).

Pharmacophore-based indirect design is a type of phenotypic drug design in which the target outcome is unknown. Reportedly, as of the end of 2020, around 89.25% of small-molecule drugs approved by the US Food and Drug Administration were derived from phenotypic discoveries (Sadri, 2023). However, the study of disease treatment with small-molecule drugs requires large numbers of experiments to elucidate the underlying pathway mechanisms and targets. Using network bioinformatics, the interactions among drugs, targets, and diseases can be integrated, helping to accelerate drug development. Network pharmacology is now widely used to explore mechanisms of disease treatment, especially for multi-component and multi-target therapies in natural medicine (Li R. et al., 2024; Li H. C. et al., 2024; Hopkins, 2008). In this paper, interrelated data networks of potential targets and molecular therapeutic mechanisms of the candidate compounds were analyzed at multiple levels using an innovative method that combines single-molecule network efficacy with network pharmacology. Furthermore, this study experimentally elucidated that one of the compounds, Z3, exerts protective antioxidant effects via the Nrf2/HO-1 pathway, thereby offering a new therapeutic strategy and potential therapeutic drug for the treatment of CIRI.

## 2 Materials and methods

### 2.1 Chemicals, consumables, and instruments

#### 2.1.1 Chemicals and consumables

All solvents and reagents were analytical-grade and were sourced from Casmar (https://www.casmart.com.cn/). Reaction progress was monitored using thin-layer chromatography under 254 nm and 365 nm ultraviolet light. Products were separated and purified by silica gel column chromatography (mesh size: 200–300). The ^1H^-nuclear magnetic resonance (NMR) and ^13^C-NMR spectra were recorded using tetramethylsilane (TMS) as an internal standard on a Brock 400 MHz spectrometer. The relative molecular mass of products was determined using mass spectrometry (MS). Purity testing of the compounds was conducted through high-performance liquid chromatography (HPLC).

#### 2.1.2 Synthesis of compounds

The synthesis of the compound was initiated by dissolving cinnamic acids with different substituents (1.1 equivalents) and carbonyldiimidazole (CDI) (1.1 equivalents) in anhydrous dichloromethane, followed by stirring at room temperature for 5 min. After adding 1.0 equivalent of benzamide and 1.5 equivalents of sodium hydride in sequence, the reaction was continued at room temperature for 2–6 h to completion, and quenched by the addition of a large amount of water. The products were extracted with dichloromethane and saturated saline, and purified by column chromatography. The ^1^H-NMR, ^13^C-NMR, and liquid chromatography-mass spectrometry (LC-MS) spectral data and HPLC results (purity: >95%) of compounds Z1–Z5 were as follows.

Compound Z1, (E)-N-(3-(3-fluorophenyl)acryloyl)nicotinamide, was obtained as a white powder, with a 62% yield and a melting point of 161.3°C–164.6°C. The ^1^H NMR (400 MHz, Chloroform-*d*) spectrum exhibited signals at δ 9.17 (singlet [s], 1H), 8.88 (doublet [d], *J* = 4.8 Hz, 2H), 8.26 (s, 1H), 7.91 (double doublet [dd]t, *J* = 15.5, 4.4 Hz, 1H), 7.79 (dd, *J* = 15.7, 4.7 Hz, 1H), 7.53 (d, *J* = 4.3 Hz, 1H), 7.41 (d, *J* = 20.1 Hz, 3H), and 7.15 (s, 1H). The ^13^C NMR (101 MHz, dimethyl sulfoxide [DMSO]-*d*_6_) spectrum showed signals at δ 166.12, 153.50, 149.76, 142.58, 137.37, 136.65, 131.52, 129.78, 124.72, 124.01, 123.12, 117.52, and 114.95. In LC-MS, the molecular ion peak was detected at m/z 270.3 (M + H)^+^.

Compound Z2, (E)-N-(3-(3-(trifluoromethyl)phenyl)acryloyl)nicotinamide, was obtained as a white powder, with a 53% yield and a melting point of 144.2°C–146.2°C. The ^1^H NMR (400 MHz, Chloroform-*d* and CDCl_3_) spectrum displayed signals at δ 9.19 (s, 1H), 8.87 (d, *J* = 27.6 Hz, 2H), 8.26 (d, *J* = 7.9 Hz, 1H), 7.98 (d, *J* = 15.8 Hz, 1H), 7.92–7.83 (m, 3H), 7.71 (d, *J* = 7.7 Hz, 1H), 7.59 (t, *J* = 7.7 Hz, 1H), and 7.54 (s, 1H). The ^13^C NMR (101 MHz, DMSO-*d*_6_) spectrum exhibited signals at δ 166.03, 153.53, 149.75, 142.08, 136.64, 136.03, 132.32, 130.71, 129.75, 127.15, 124.97, 124.01, and 123.69. In LC-MS, a molecular ion peak was observed at m/z 320.3 (M + H)^+^.

Compound Z3, (E)-N-(3-(3-bromophenyl)acryloyl)nicotinamide, was obtained as a white powder, with 68% yield and a melting point of 144.5°C–147.6°C. The ^1^H NMR (400 MHz, DMSO-*d*_6_ and CDCl_3_) spectrum displayed signals at δ 9.22 (s, 2H), 8.89 (d, *J* = 4.0 Hz, 1H), 8.29 (d, *J* = 7.9 Hz, 1H), 7.92–7.75 (m, 3H), 7.63–7.57 (m, 2H), 7.54 (dd, *J* = 7.5, 5.2 Hz, 1H), 7.33 (t, *J* = 7.9 Hz, 1H). The^13^C NMR (101 MHz, DMSO-*d6)* spectrum exhibited signals at δ 166.07, 153.53, 149.75, 142.20, 137.39, 136.65, 133.44, 131.68, 131.09, 129.77, 127.52, 124.02, 123.26, and 122.84. In LC-MS, a molecular ion peak was observed at m/z 331.1 (M + H)^+^.

Compound Z4, (E)-N-(3-(3,4-dichlorophenyl)acryloyl)nicotinamide, was obtained as a white powder, with 58% yield and a melting point of 215.4°C–217.2°C. The ^1^H NMR (400 MHz, DMSO-*d*_6_ and DMSO) spectrum displayed signals at δ 11.37 (s, 1H), 9.09 (d, *J* = 1.8 Hz, 1H), 8.81 (dd, *J* = 4.8, 1.5 Hz, 1H), 8.29 (dt, *J* = 8.0, 1.9 Hz, 1H), 7.97 (d, *J* = 1.7 Hz, 1H), 7.78–7.67 (m, 3H), 7.59 (dd, *J* = 7.9, 4.8 Hz, 1H), and 7.35 (d, *J* = 15.8 Hz, 1H). The ^13^C NMR (101 MHz, DMSO-*d*_6_) spectrum exhibited signals at δ 166.09, 153.54, 149.76, 141.14, 136.64, 135.76, 133.11, 132.32, 131.72, 130.49, 129.72, 128.30, and 123.93. In LC-MS, a molecular ion peak was observed at m/z 321.2 (M + H)^+^.

Compound Z5, (E)-N-(3-(4-methoxyphenyl)acryloyl)nicotinamide, was obtained as a white powder, with 56% yield and a melting point of 183.2°C–185.1°C. The ^1^H NMR (400 MHz, DMSO-*d*_6_ and DMSO) spectrum displayed signals at δ 11.25 (s, 1H), 9.07 (s, 1H), 8.81 (d, *J* = 3.3 Hz, 1H), 8.28 (d, *J* = 7.5 Hz, 1H), 7.77–7.53 (m, 4H), 7.19 (d, *J* = 15.7 Hz, 1H), 7.05 (d, *J* = 8.0 Hz, 2H), and 3.82 (d, *J* = 12.3 Hz, 3H). The ^13^C NMR (101 MHz, DMSO-*d*_6_) spectrum exhibited signals at δ 166.43, 161.73, 153.37, 149.72, 144.23, 136.60, 130.51, 130.00, 129.46, 127.43, 123.97, 118.70, 115.06, and 55.89. In LC-MS, a molecular ion peak was observed at m/z 282.4 (M + H)^+^.

### 2.2 Prediction of physical and chemical properties of the compounds

Next, we sought to evaluate the potential blood–brain barrier (BBB) permeability of the compounds as neurodrugs. Employing ChemDraw software, we obtained the following physicochemical properties of Z1–Z5: relative molecular weight (MW), number of hydrogen bond donors (HBDs), number of hydrogen bond acceptors (HBAs), calculated lipoid–water partition coefficient (ClogP), and topologically polarized surface area (TPSA).

### 2.3 Screening of target compounds and CIRI target genes

The potential targets of the candidate compounds were predicted and de-screened through the PharmMapper and SwissTargetPrediction databases based on structural similarity (Probability >0.7). Taking “cerebral ischemia–reperfusion injury” as the target term, potential targets related to CIRI were screened through the GeneCards online database (https://www.genec-ards.org/), using a score of >2.0 as the selection criterion. The UniProt database (https://www.uniprot.org/) was then used to convert the target information into specific protein ID numbers, and corresponding gene names were found in the GeneCards database. A Venn diagram was drawn using VennDiagram software to obtain the intersection of the candidate compounds with the potential targets of CIRI-related disease.

### 2.4 Construction of a protein–protein interaction (PPI) network of core target genes

The intersecting genes were imported into the STRING database to complete the construction of the PPI network, hide the free target genes in the network, and export the related information file. Analysis and mapping of core target genes in the PPI network were undertaken using Cytoscape software. Finally, Geno Ontology (GO) and Kyoto Encyclopedia of Genes and Genomes (KEGG) enrichment analyses were performed on the target genes, from which the histogram and bubble diagram were drawn.

### 2.5 Molecular docking and scoring

The structure of each core target protein was obtained from the UniProt database. The Protein Data Bank coding or AlphaFold modeling accession numbers for the protein structures of the human STAT1, NFKB1, NFEL2R2, ITGB1, NR3C1, JNK3 and PIK3R1 proteins are as follows: AF-P42224-F1, 1SVC, 2FLU, 4WK0, AF-P04150-F1 and AF-P27986-F, respectively. Small molecule 3D ligands were subjected to energy minimization using Chem3D after being drawn with Chemdraw. Autodock software tools were employed to remove ligands and water molecules from each protein structure. Briefly, the Sitemap module was used to determine the docking box center, after which molecular docking was performed using ligand docking to obtain the docking fraction. The docking results were visualized using PyMOL.

### 2.6 Reagents, culture medium, and antibodies for biological experiments

Hydrogen peroxide (H_2_O_2_; Cat# H112515) and tert-butylhydroquinone (TBHQ; Cat# B105352) were purchased from Aladdin (Shanghai, China). Zinc protoporphyrin (ZnPP; Cat# HY-101193) was from MedChemExpress (Monmouth Junction, NJ, United States), and ED (Cat# 443300) and 2,3,5-triphenyltetrazolium (TTC; Cat# T8877) were from Sigma-Aldrich (St. Louis, MO, United States). Dulbecco’s modified Eagle’s medium (DMEM; Cat# C11995500BT), fetal bovine serum (FBS; Cat# 10270-106), and trypsin (Cat# 25200-056) were from Gibco/Invitroge. The malondialdehyde (MDA) assay kit (Cat# S0131S) and dichloro-dihydrofluorescein diacetate (DCFH-DA) probe kit (Cat# S0033S) were from Beyotime Institute of Biotechnology (Nanjing, China). The anti-sestrin 2 (Cat# 10795-1-AP), anti-HO-1 (Cat# 10701-1-AP), anti-GAPDH (Cat# 10494-1-AP), and anti-rabbit IgG (Cat# SA00001-2) antibodies were from Proteintech (Wuhan, China). The anti-p62 (Cat# sc-48402) and anti-Nrf2 (Cat# sc-365949) antibodies were from Santa Cruz Biotechnology (Santa Cruz, CA, United States), and the anti-mouse IgG (Cat# 7076S) antibody was from Cell Signaling Technology (Danvers, MA, United States).

#### 2.6.1 Cell culture

The PC12 rat pheochromocytoma cell line was from the Cell Storage Center of Wuhan University (Wuhan, China). The cells were cultured in growth medium comprising DMEM, 10% FBS, and 0.4% penicillin-streptomycin solution (10,000 U/mL penicillin and 10 mg/mL streptomycin) in a 37°C incubator with 5% CO_2_ (Thermo Fisher Scientific, Waltham, MA, United States).

#### 2.6.2 MTT assay

PC12 cells were seeded into 96-well plates (5 × 10^3^ cells/well). The cells were pretreated with Z1–Z5 for 18/24 h, followed by 500 μM H_2_O_2_ for 24 h. Next, each well received 20 µL of 3-(4,5-dimethylthiazol-2-yl)-2,5-diphenyltetrazolium bromide (MTT) solution at a concentration of 5 mg·mL^−1^, followed by incubation in the dark for 4 h. Thereafter, the medium was carefully removed, and 120 µL of DMSO was added to dissolve the formazan crystals. The optical density was subsequently measured at 490 nm using a microplate reader (Molecular Devices, United States).

#### 2.6.3 Colony formation assay

PC12 cells (1000 cells/mL) were transferred to 6-well plates for 24 h, treated with Z3 (0.625, 1.24, 2.5 μmol·L^−1^) for another 24 h, and then exposed to 270 μmol·L^−1^ H_2_O_2_ or DMSO for an additional 5 days. Each well was emptied, washed with phosphate-buffered saline (PBS), and colonies were fixed with 4% paraformaldehyde for 15 min. After staining the cells with crystal violet, the wells were washed with PBS to remove excess stain, and the colonies were dried and photographed.

#### 2.6.4 Cell morphology observation

PC12 cells were seeded in 6-well plates (3 × 10^5^ cells/well), pretreated with Z3 (1.25, 2.5, 5, 10 μmol·L^−1^) for 24 h, then stimulated by the addition of H_2_O_2_ (1.25 mmol·L^−1^) and cultivated for a further 3 h. Cell morphology was observed and captured using an inverted microscope (Nikon, Japan).

#### 2.6.5 ROS assay

PC12 cells were seeded into 6-well plates (3 × 10^5^ cells/well). The next day, the cells were pre-incubated with Z3 (1.25, 2.5, 5, 10 μmol·L^−1^) for 24 h, followed by the addition of 1.25 mmol·L^−1^ H_2_O_2_ for another 2 h. After incubation with 1 µL DCFH-DA (10 μmol·L^−1^) for 30 min at 37°C in the dark, residual probe was removed from each sample by washing with PBS. Images were obtained with a fluorescence microscope (Nikon).

#### 2.6.6 MDA assay

PC12 cells were used to inoculate 6-well plates (3 × 10^5^ cells/well) and cultured for 24 h. The cells were then incubated with Z3 (1.25, 2.5, 5, 10 μmol·L^−1^) for 24 h, followed by stimulation with H_2_O_2_ (1.25 mmol·L^−1^) for 3 h. The cells were harvested and the concentration of protein was determined. MDA levels were detected using the thiobarbituric acid method, in accordance with the MDA assay kit instructions.

#### 2.6.7 Immunofluorescence

PC12 cells were seeded into 6-well plates (8 × 10^4^ cells/well). After attachment, the cells were exposed to Z3 (10 μmol·L^−1^) or TBHQ (10 μmol·L^−1^) for stipulated periods and then washed with PBS. Subsequently, the cells were fixed in 4% paraformaldehyde for 20 min, permeabilized with 1% Triton X-100 for 15 min, and blocked in 2% BSA for 1 h. The cells were then incubated with a primary antibody at 4°C overnight, followed by a fluorescence-conjugated secondary antibody at 37°C for 1 h in the dark, and then stained with DAPI at room temperature for 8 min in the dark. Images were acquired under a fluorescence microscope (Nikon).

#### 2.6.8 Western blotting

Total protein extracts from treated PC12 cells were prepared using RIPA lysis buffer. Following centrifugation, protein concentrations were quantified using Coomassie brilliant blue staining (Bio-Rad, Shanghai, China). Proteins were subsequently resolved by 10% sodium dodecyl sulfate-polyacrylamide gel electrophoresis and transferred onto polyvinylidene fluoride membranes (Millipore, MA, United States). The membranes were blocked with 5% skim milk, washed with tris-buffered saline containing Tween-20, and incubated overnight at 4°C with the following primary antibodies: anti-sestrin 2 (dilution 1:1000), anti-p62 (1:1000), anti-HO-1 (1:1000), anti-Nrf2 (1:1000), and anti-GAPDH (1:1000). On the following day, the membranes were washed and incubated with secondary antibodies for 1 h at room temperature. Visualization of target proteins was achieved using a Gel Imaging Analysis System (Bio-Rad, Hercules, CA, United States). ImageJ software (National Institutes of Health [NIH], Bethesda, MD, United States) was employed for subsequent analysis.

#### 2.6.9 HO-1 inhibitor experiment

PC12 cells were cultured in 96-well plates (5 × 10^3^ cells/well). At 24 h post seeding, cells were pretreated with ZnPP (15 μmol·L^−1^) for 1 h prior to treatment with Z3 at a range of concentrations (1.25, 2.5, 5, and 10 μmol·L^−1^) for another 24 h. Finally, the cells were exposed to H_2_O_2_ (500 μmol·L^−1^) for 24 h and then tested for cell viability via the MTT assay.

#### 2.6.10 LDH assay

Lactate dehydrogenase (LDH), a cytoplasmic enzyme, is released into the extracellular space upon cell membrane damage or cell death. PC12 cells were plated in 96-well plates at 5 × 10^3^ cells/well. After plating, cells were maintained in starvation medium and pretreated with Z3 for 18 h. They were then exposed to H_2_O_2_ for 24 h to induce oxidative damage. The release of LDH was measured using the LDH Release Assay Kit.

#### 2.6.11 Animals

Thirty wild-type C57BL/6 male mice (bodyweight, 20–25 g) were obtained from the Animal Center of Wenzhou Medical University (Wenzhou, China). All mice were fed in a room with controlled temperature (24°C) and lighting (12 h/day). The animal experiments were approved by the Wenzhou Medical University Animal Policy and Welfare Committee, and were carried out in accordance with the standards of NIH Publication No. 8023.

#### 2.6.12 Middle cerebral artery occlusion (MCAO) modeling

The mice were randomly divided into the following five groups (n = 6/group): Sham operation (Sham group), normal saline + model (NS group), vehicle (cremophor: normal saline = 1:4) + model (Vehicle group), model + Z3 (15 mg·kg^−1^, Z3 group), and model + ED (15 mg⋅kg^−1^, ED group). Drugs were administered via intraperitoneal injection 2 h prior to the MCAO procedure. Anesthesia was induced using 1% sodium pentobarbital (50 mg·kg^−1^). During the MCAO procedure, a minor cervical incision was performed on anesthetized mice to expose the right common carotid artery, internal carotid artery (ICA), and external carotid artery (ECA). Subsequently, a small incision was made on the ECA to facilitate the insertion of an occlusion line into the ICA, thereby inducing ischemia. After 1.5 h, the occlusion suture was withdrawn to allow for 48 h of reperfusion. The Sham group underwent identical procedural steps, excluding the insertion of the suture.

#### 2.6.13 TTC staining

Following 48 h of reperfusion, TTC staining was performed. TTC staining was used to quantitatively assesses cerebral infarction areas. Mouse brains were frozen at −20°C for 20 min, sliced into five sections, stained with 2% TTC at 37°C for 20 min, fixed with 4% paraformaldehyde, photographed, and analyzed using ImageJ software.

#### 2.6.14 Neurobehavioral scoring

Neurological deficit score was evaluated at 48 h after reperfusion. Mice with stroke were evaluated and assigned a neurological score using the Longa scale, as follows: 0, no neurological deficits; 1, cannot fully extend the contralateral front claw; 2, circles to the opposite side of the cerebral infarction; 3, falls down to the opposite side of the cerebral infarction; 4, unable to walk and loss of consciousness. As the score increased, the dyskinesia became more severe.

#### 2.6.15 Statistical analysis

Data in this paper are expressed as means ± standard deviation (SD). GraphPad Prism 9.0 (GraphPad, San Diego, CA, United States) was used for data analysis. Statistical significance between groups was determined by Student’s t-test or one-way analysis of variance (ANOVA). A *P* value <0.05 was considered to represent a statistically significant difference.

## 3 Results

### 3.1 Synthesis of novel diimides Z1–Z5

**SCHEME 1:**
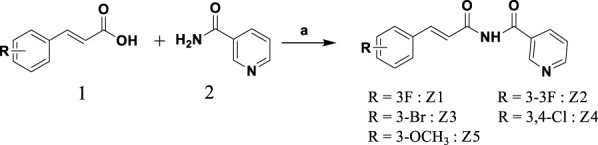
Synthesis and structure of novel diimide derivatives Z1–Z5. (a) CDI, NaH, DCM, r.t.

On the basis of previously explored acid and amide condensation conditions (Zheng et al., 2023a), compounds Z1–Z5 were synthesized as shown in Scheme 1. Z1–Z5 were obtained by reacting 1.1 equivalents of acid with 1.0 equivalent each of amide, CDI, and sodium hydride (NaH) as condensing agents, and dichloromethane (DCM) as solvent. Each compound was characterized by NMR and MS, with a good yield and a purity >95%.

### 3.2 Physical and chemical properties of the candidate compounds

The BBB is an important limiting factor in the development of drugs to treat brain diseases. Lipinski et al. summarized the five physicochemical properties of proprietary drugs that influence absorption and permeability, while Sun’s team further proposed strategies to improve BBB penetration, such as increasing lipophilicity and reducing the polar atomic surface area of the drug molecule. Therefore, we used ChemDraw to forecast relevant indicators for the synthesized compounds (Table 1). The relative MW (<500 Da), number of HBDs (<5), and number of HBDs (<10) of Z1–Z5 all adhered to Lipinski’s rule of five, suggesting favorable drug-like properties for each. The ClogP values over a period of 2–3 days indicated that the compounds had both lipophilic and hydrophilic properties, with good biofilm penetration alongside suitable solubility. Studies have shown that TPSA values <78 are conducive to brain penetration. Table 1 shows that the TPSAs of all five compounds were determined to be <68, aligning with the ideal design for a neurodrug. These results are also consistent with the predicted results of the SwissADME network, indicating that this type of compound theoretically has good permeability in the blood-brain barrier and deserves further in-depth exploration. Computation-based statistical methods offer empirical guidelines for compound design, greatly accelerating the development of targeted drugs. However, the physicochemical properties of these compounds require further validation in preclinical evaluation studies.

**TABLE 1:** Physical and chemical properties of Z1–Z5.

Compd	MW	HBD	HBA	CLogP	tPSA
Z1	270.08	1	3	3.04	58.53
Z2	320.27	1	3	3.78	58.53
Z3	331.17	1	3	3.76	58.53
Z4	320.01	1	3	4.02	58.53
Z5	282.30	1	4	2.82	67.76

### 3.3 Compound Z3-mediated amelioration of H_2_O_2_-induced damage in PC12 cells

**FIGURE 2:**
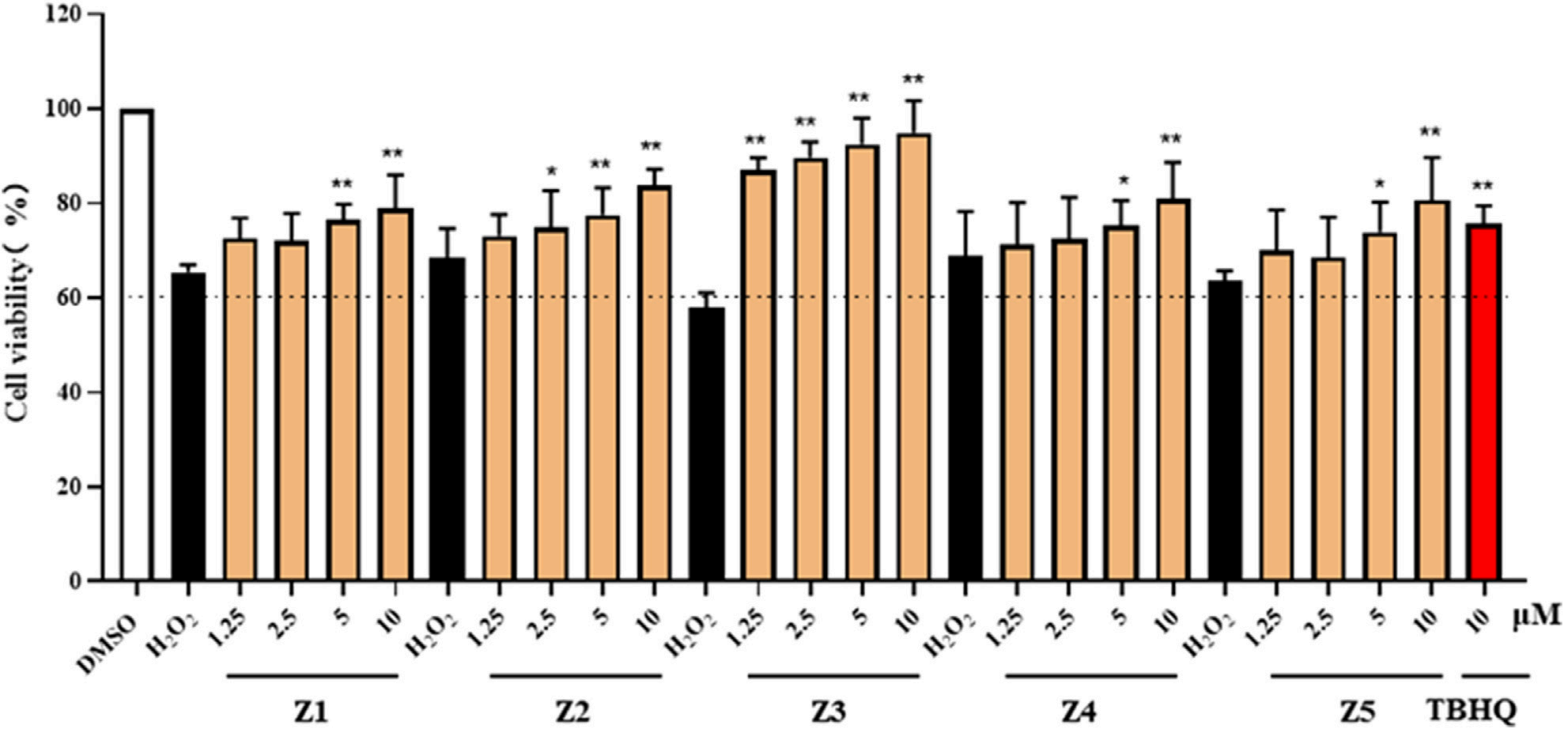
Protective antioxidant effect of the Z1–Z5 family of compounds. MTT assays of cell viability of PC12 cells grown for 1 day on 96-well plates, treated with the indicated concentrations of each candidate compound for 18 h, and stimulated with H_2_O_2_ (500 μM) for 24 h; *P < 0.05, **P < 0.01.

PC12 cells exposed to H_2_O_2_ undergo a series of physiological and pathological alterations that closely mimic the pathophysiological processes of CIRI, including oxidative stress, inflammation, apoptosis, and autophagy. Therefore, this cell model may help elucidate the pathophysiological process of CIRI to facilitate the discovery of new therapeutic approaches or drugs. Here, we pre-incubated PC12 cells with different concentrations of Z1–Z5 for 18 h, and then stimulated the cells with 500 μM H_2_O_2_ for a further 24 h. MTT assay results showed that all five compounds increased cell survival in a concentration-dependent manner compared with H_2_O_2_ group. However, Z3 was the most protective of the five compounds, performing significantly better than the positive control drug TBHQ (Figure 2). Therefore, we selected Z3 for further study of its protective antioxidant effect.

**FIGURE 3:**
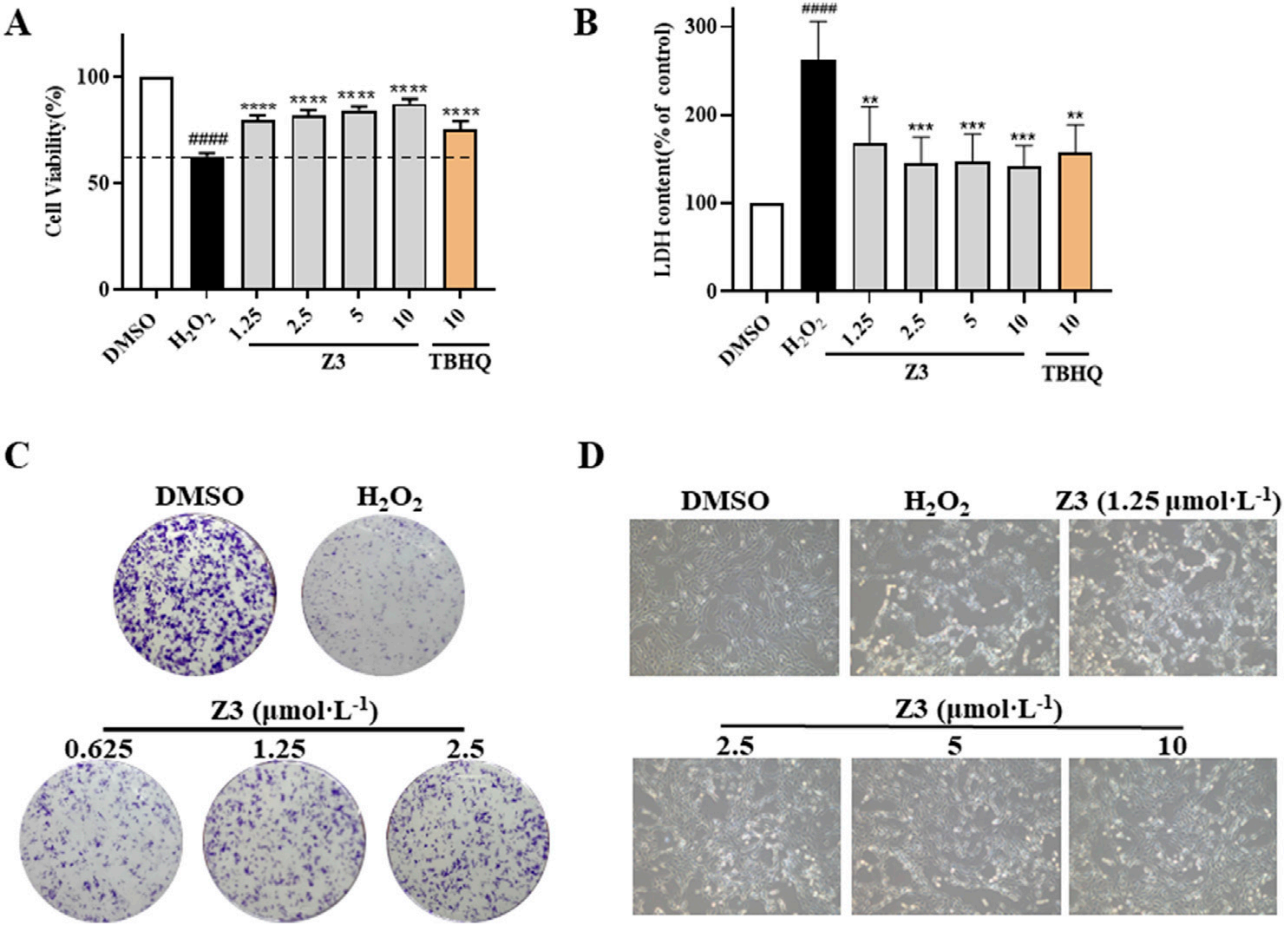
Cytoprotective effects of Z3 against H_2_O_2_-induced PC12 cell damage. **(A,B)** MTT **(A)** and LDH **(B)** assays to detect the cytoprotective effect of Z3 against H_2_O_2_-induced PC12 cells treated with Z3, TBHQ for 24 h, then exposed to H_2_O_2_ for another 24 h. **(C)** Colony formation assay of PC12 cells pretreated with Z3 for 24 h, and then exposed to H_2_O_2_. Cells were stained with crystal violet. **(D)** Inverted microscopy showing the morphological improvements in crystal violet-stained PC12 cells pretreated with Z3, then stimulated with H_2_O_2_ compared with DMSO-treated cells. Data represented as means ± SD (n = 3); ^####^ *P* < 0.0001; **P < 0.01, ***P < 0.001.

Next, MTT and LDH assays were used to evaluate Z3 for cytoprotection of PC12 cells against H_2_O_2_ stimulation. Treatment with Z3 (1.25–10 μmol·L^−1^) for 24 h or 18 h did not affect the survival rate of cells (Figures 3A,B). Meanwhile, compared with the blank group, compound Z3 had no cytotoxic effect on P12 cells at concentrations of 1.25, 2.5, 5, and 10 μmol·L^−1^ (Supplementary Figure S1). In colony formation assays, stimulation with H_2_O_2_ significantly inhibited the formation of cell colonies compared with DMSO. Pre-treatment with Z3 (0.625, 1.25, and 2.5 μmol·L^−1^) increased the formation of cell colonies in a concentration-dependent manner (Figure 3C). Under inverted microscopy, H_2_O_2_-stimulated cells showed shrinkage and the loss of synapses. These cell status parameters were improved by pretreatment with Z3 (1.25, 2.5, 5, and 10 μmol·L^−1^) in a concentration-dependent manner (Figure 3D). These findings demonstrated that, under exposure to H_2_O_2_, PC12 cell survival was significantly enhanced by pretreatment with Z3.

### 3.4 Network of common and core target genes of Z3 and CIRI

**FIGURE 4:**
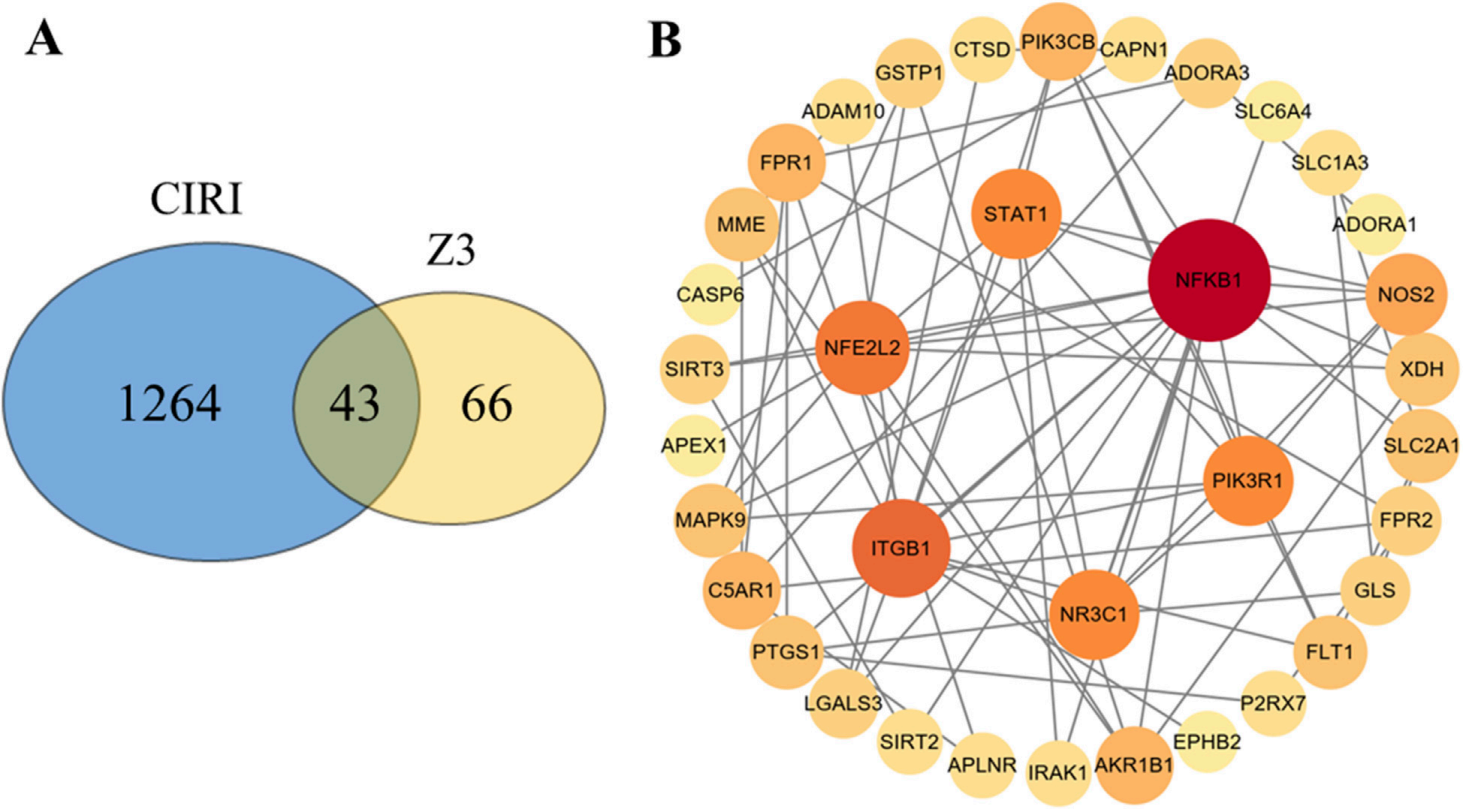
Venn diagram **(A)** and PPI action network diagram **(B)** of common gene targets between Z3 and active components of CIRI.

The application of bionetwork informatics can accelerate the research and development of drugs, playing a guiding role in target identification and therapeutic mechanisms. In this study, we combined single-molecule network target prediction with network pharmacological analysis. By intersecting 1307 CIRI targets and 109 candidate drug targets, we identified 43 common targets associated with Z3-mediated protection of CIRI, as illustrated by a Venn diagram (Figure 4A). These common targets were then uploaded to the STRING database, and a PPI network was constructed using Cytoscape, resulting in the identification of 43 core target genes for Z3-mediated protection against CIRI. These included *NFE2L2* (also known as *Nrf2*), *NR3C1*, *PIK3R1*, *ITGB1*, *STAT1*, and *NFKB1* (Figure 4B).

### 3.5 GO enrichment analysis of common target genes

**FIGURE 5:**
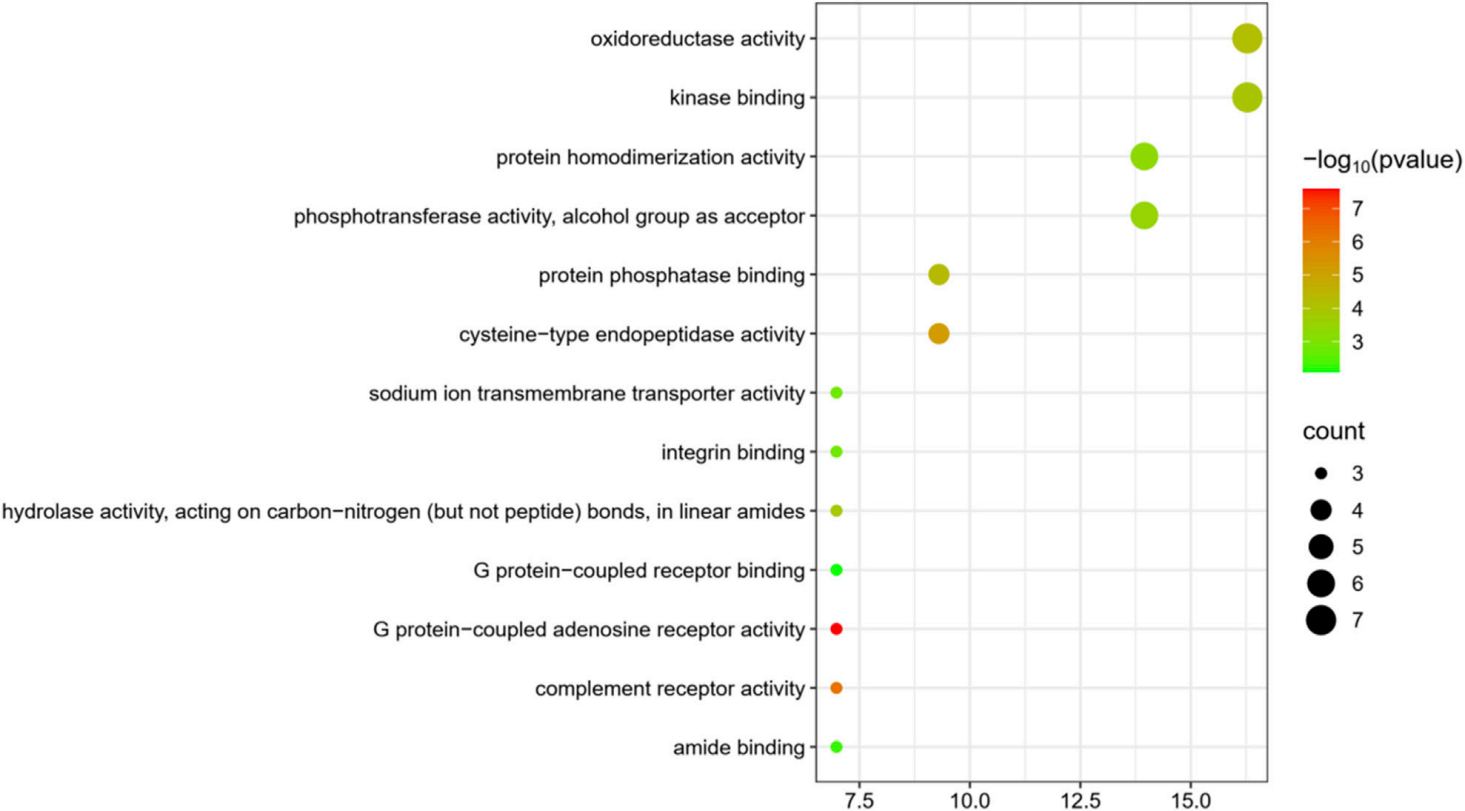
Results of GO enrichment analysis of common target genes between Z3 and CIRI. The top 13 biological processes related to CIRI are shown.

GO enrichment analysis of the 43 common target genes indicated that Z3 protects the top 13 biological processes involved in CIRI, including oxidation–reduction (REDOX) enzyme activity, kinase binding, protein homologous dimerization activity, phosphotransferase activity, protein phosphorylation binding, cysteine-type endopeptidase activity, and sodium ion transmembrane transporter activity (Figure 5). Oxidoreductase activity had the strongest correlation with CIRI, indicating that Z3 protect cells from oxidative stress damage by regulating the REDOX state. Therefore, we continued to investigate these antioxidant properties with further experiments.

### 3.6 Z3-mediated reduction of H_2_O_2_-induced oxidative stress damage in PC12 cells

**FIGURE 6:**
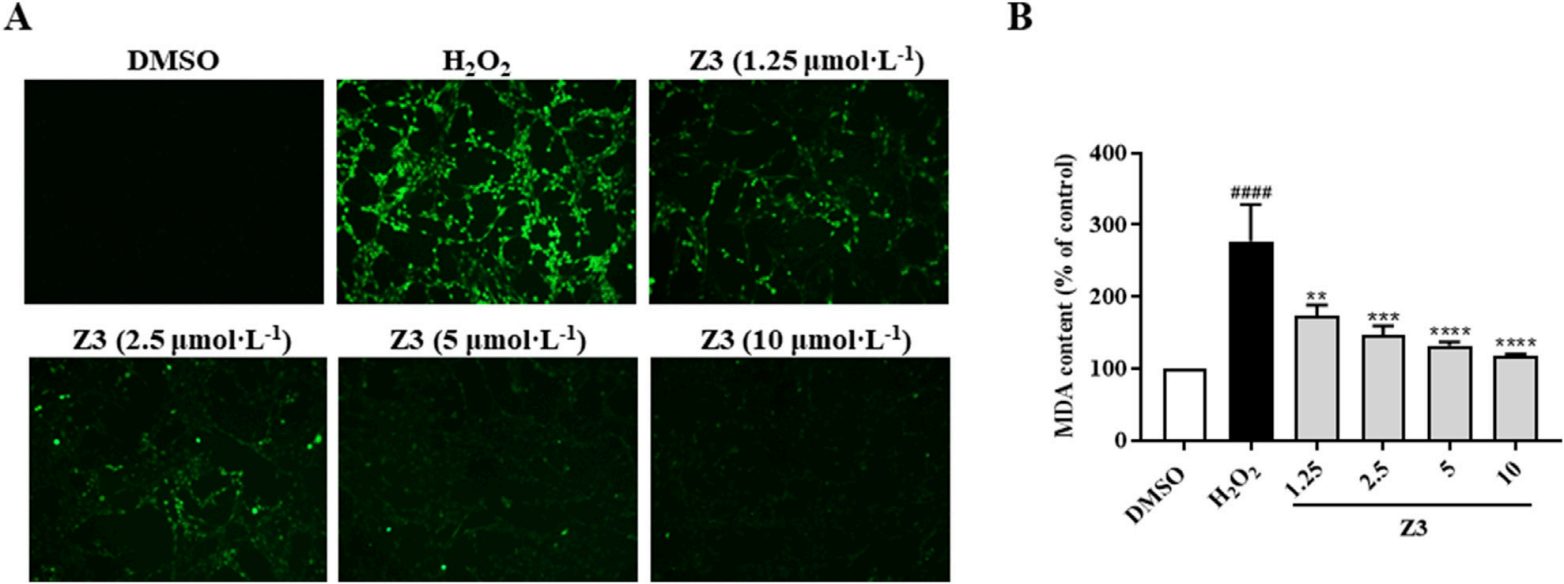
Antioxidant activity of Z3 in PC12 cells. **(A)** Representative fluorescence images showing the reduction in the accumulation of intracellular ROS in cells pretreated with Z3 or DMSO control for 24 h, then stimulated with H_2_O_2_ for 2 h, followed by incubation with DCFH-DA for 30 min. **(B)** Reduction in MDA in cells pretreated with Z3 or DMSO for 24 h, then stimulated with H_2_O_2_ for 3 h. Data expressed as means ± SD (n = 3); ^####^ *P* < 0.0001; ^**^ *P* < 0.01, ^***^ *P* < 0.001, ^****^ *P* < 0.0001.

H_2_O_2_ is a strong oxidizing molecule that can cause oxidative stress reactions, leading to damage to cell membranes, proteins, DNA, and other molecules. Therefore, we used the classic H_2_O_2_-induced PC12 cell injury model to study antioxidant activity, with relevance to CIRI (Lv et al., 2017). Research has shown that excessive ROS levels during oxidative stress can lead to an increase in the lipid peroxidation product MDA, thus ROS and MDA serve as markers of oxidative stress. As shown in Figure 6, cells pre-incubated with compound Z3 (1.25, 2.5, 5, and 10 μmol·L^−1^) for 24 h prior to stimulation with 1.25 mmol·L^−1^ H_2_O_2_. Z3 showed concentration-dependent reductions in ROS and MDA production, indicating that Z3 can effectively reduce oxidative stress damage *in vitro*.

### 3.7 Computer-simulated molecular docking of Z3 with core proteins

**FIGURE 7:**
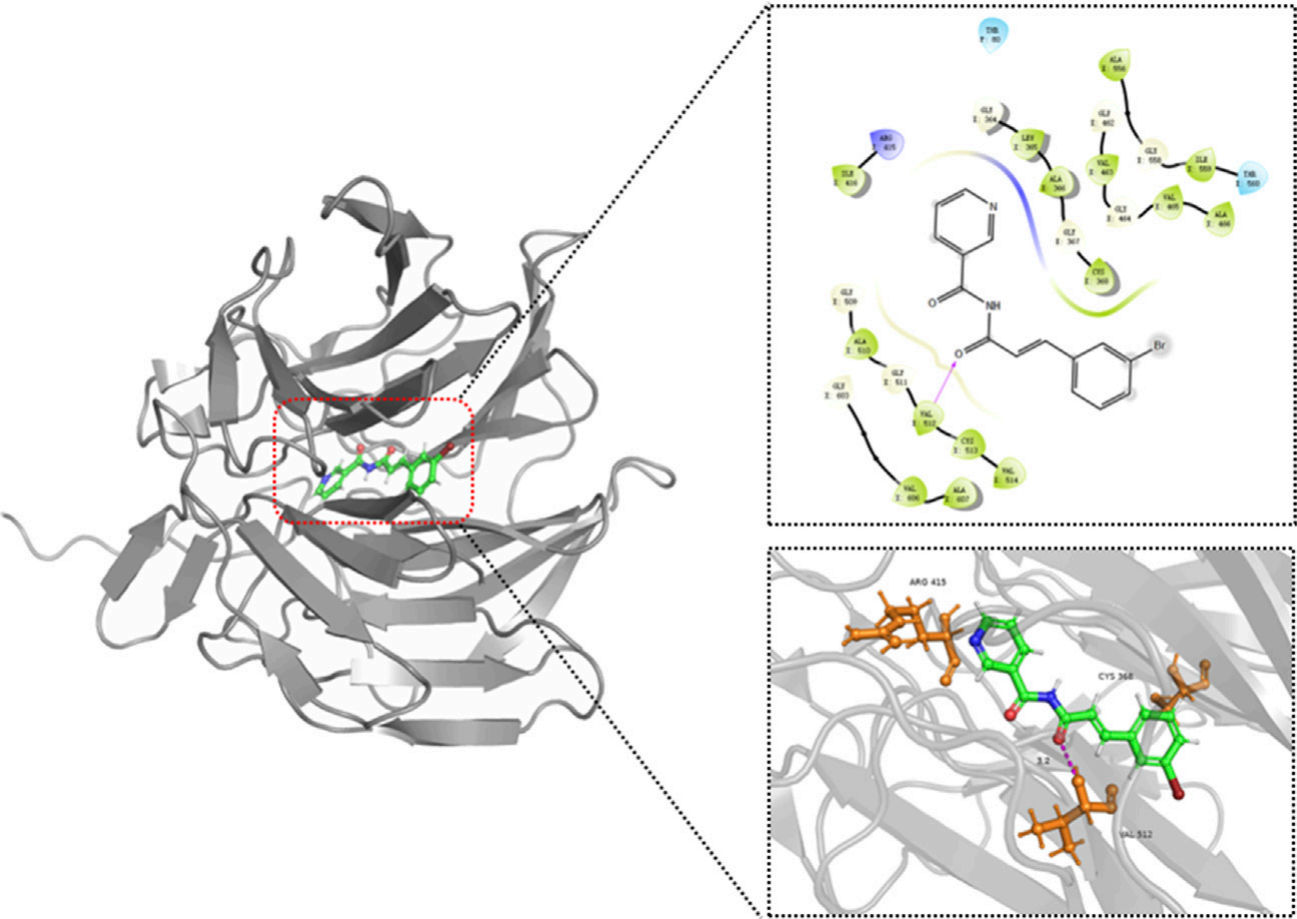
Molecular docking diagrams of Z3 with Nrf2 (PDB:2FLU).

To continue exploring the signaling pathways related to Z3-targeted proteins, we used computer-simulated molecular docking to score the proteins encoded by core target genes. Among these, NFE2L2 (Nrf2) had the best docking results (Table 2). Further molecular docking analysis showed that the pyridine ring of Z3 penetrated into the Nrf2 pocket, with the Z3 carbonyl group forming a stable hydrogen bond with valine 512 in Nrf2, resulting in good binding (Figure 7). Nrf2 is a key transcription factor in the Nrf2/HO-1 pathway, which plays an important role in regulating the cellular antioxidant response involved in protecting against CIRI. Therefore, we focused on the role of compound Z3 in the Nrf2/HO-1 pathway.

**TABLE 2:** Molecular docking scores of the Z3-targeted proteins encoded by the seven core genes.

Protein	NFEL2L2	NR3C1	PIK3R1	ITGB1	STAT1	NFKB1
Docking Score	−6.72	−6.43	−6.01	−5.23	−5.13	-2.94

### 3.8 Z3-mediated activation of the sestrin 2/p62/Nrf2/HO-1 signaling axis

**FIGURE 8:**
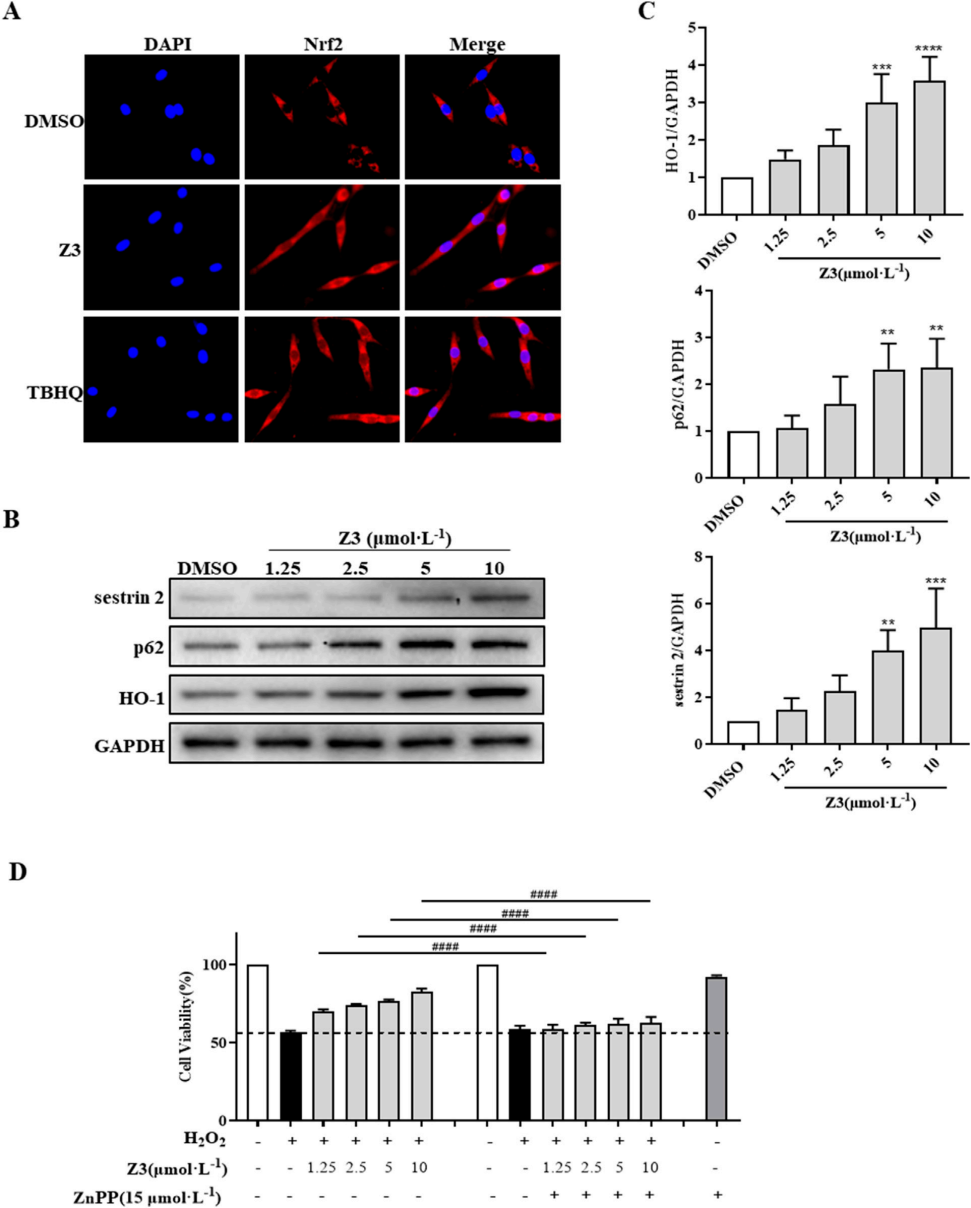
Z3-mediated activation of the sestrin 2/p62/Nrf2/HO-1 signaling pathway in PC12 cells. **(A)** Immunofluorescence of cells pre-incubated with 10 μmol·L^−1^ Z3 or TBHQ for 6 h and stained with Nrf2 antibody (red) and DAPI (blue), showing the nuclear translocation of Nrf2. **(B,C)** Western blotting of sestrin 2, p62, and HO-1 in cells treated with Z3 for 24 h **(D)** MTT viability assays of cells pretreated with ZnPP for 1 h prior to the addition of Z3 and stimulation with H_2_O_2_. Data expressed as means ± SD (n = 3); ^####^ *P* < 0.0001; ^**^ *P* < 0.01, ^***^ *P* < 0.001, ^****^ *P* < 0.0001.

Previous research has demonstrated the protective effects of Nrf2/HO-1 pathway agonists in CIRI. To characterize the correlation between the antioxidant activity of Z3 and the Nrf2/HO-1 pathway, we used immunofluorescence staining to determine whether Z3 can promote the transfer of Nrf2 from the cytoplasm to the nucleus in PC12 cells. The results showed that Nrf2 (red fluorescence) was distributed around the nucleus (blue fluorescence) in the DMSO control group, with little overlap between the two, indicating that Nrf2 was mainly distributed in the cytoplasm (Figure 8A). Compared with the DMSO group, the Z3 and TBHQ treatment groups exhibited some overlap between Nrf2 and the nucleus, indicating an increase in the presence of Nrf2 in the nucleus. These results indicated that Z3 induces nuclear translocation of Nrf2.

Next, Western blot analysis was used to investigate the effect of Z3 on the protein expression of downstream target HO-1 in the Nrf2 pathway. The results showed that Z3 increased HO-1 protein expression in a concentration-dependent manner in PC12 cells (Figures 8B,C). Next, specific inhibition of HO-1 by ZnPP was used to verify whether Z3-activated HO-1 expression has a protective effect on H_2_O_2_-induced oxidative damage. As shown in Figure 8D, cells treated with ZnPP alone showed no significant decrease in viability compared with untreated cells. Compared with H_2_O_2_-induced cells, cells pretreated with Z3 (1.25–10 μmol·L^−1^) showed an approximately 10%–25% increase in viability. However, when cells were simultaneously treated with Z3 (1.25–10 μmol·L^−1^) and ZnPP, the cell survival rate dropped to a level close to that of cells induced with H_2_O_2_ alone. In summary, Z3 exerted a protective effect against H_2_O_2_-induced oxidative damage through the Nrf2/HO-1 signaling pathway.

To investigate the upstream mechanism of Z3-mediated activation of Nrf2/HO-1, the effect of Z3 on protein expression of sestrin 2 and p62 was analyzed using Western blotting. The results showed that Z3 significantly increased the expression of both proteins in a concentration-dependent manner, with the highest increase observed at 10 μmol·L^−1^ (Figures 8B,C).

### 3.9 Z3-mediated alleviation of MCAO-induced CIRI

**FIGURE 9:**
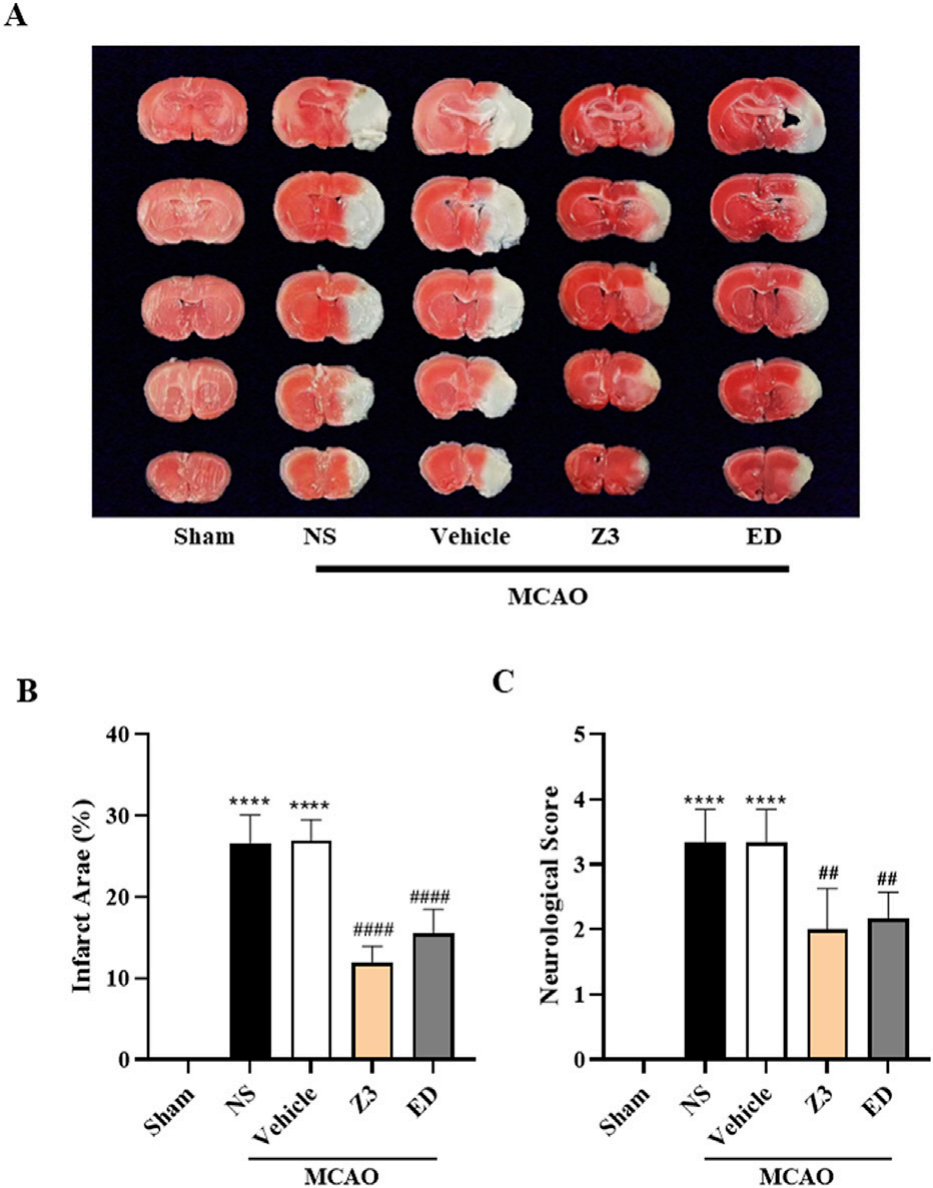
Protective effect of Z3 on MCAO-induced CIRI. **(A)** TTC staining analysis of representative brain slices from model mice treated with 15 mg·kg^−1^ Z3. **(B)** Quantitative analysis of brain infarction was performed by calculating the ratio of infarct area to whole brain area in individual mice. **(C)** Quantitative analysis of neurobehavioral scores in MCAO mice with intraperitoneal injection of normal saline (NS), vehicle, Z3, or ED. Data expressed as means ± SD (n = 5–8); ^##^ *P* < 0.01, ^####^ *P* < 0.0001; ^****^ *P* < 0.0001.

To investigate the neuroprotective effect of Z3 *in vivo*, CIRI model mice induced by MCAO were employed for TTC staining analysis of the area of cerebral infarction and Longa scoring of neurological activity. The conventional positive control ED was administered intraperitoneally at a concentration of 15 mg·kg^−1^. To investigate the effect of Z3 *in vivo*, we also set its concentration to 15 mg·kg^−1^. TTC staining of brain tissues of MCAO-induced CIRI mice revealed magenta-red coloration in normal tissues and no staining (pale white appearance) in infarcted regions. As shown in Figures 9A,B, the Sham group showed no significant infarct damage, whereas both the NS and Vehicle groups exhibited severe cerebral infarction, with infarct areas reaching 25%. Notably, both the Z3 and ED treatment groups showed significant reductions in infarct size, with superior therapeutic efficacy in the Z3-treated group (11.9% vs 15.5% infarct area). In addition, the neurobehavioral deficit scores were significantly decreased in model mice treated with Z3 or ED compared with (Figure 9C). In summary, Z3 had good therapeutic effects on MCAO-induced CIRI mice.

## 4 Discussion

The mechanisms underlying CIRI are extremely complex, encompassing calcium overload, oxidative stress, inflammatory responses, mitochondrial damage, and excitotoxicity of amino acids, among others (Shao et al., 2018; Ran et al., 2021). Among them, oxidative stress has been proven to be a key factor associated with the onset and progression of CIRI (Liu et al., 2020; Wang et al., 2019). Oxidative stress arises from an imbalance between the oxidation and antioxidant systems, resulting in excessive production of ROS that overwhelm intracellular antioxidant defense systems, causing irreversible damage to DNA, proteins, membranes, and lipids, and ultimately leading to apoptotic cell death (Polianskyte-Prause et al., 2022). Antioxidants can eliminate ROS by initiating complex biological processes and activating endogenous antioxidant enzymes. Therefore, supplementation with exogenous antioxidants is considered an effective treatment strategy for CIRI (Shen et al., 2022). To date, despite the large number of known antioxidants, only one has been used in the treatment of CIRI: the free radical scavenger ED (Singh et al., 2019). In 2020, the edaravone dexborneol formulation was launched, leveraging the free radical scavenging activity of ED and the anti-inflammatory properties of dexborneol to synergistically improve neurological function in acute ischemic stroke (Xu et al., 2019). The development of such novel and highly effective antioxidants holds great significance for the prevention and treatment of CIRI.

The protective effects of Nrf2 agonist-type antioxidants have made them the most widely researched antioxidants for CIRI. Activated Nrf2 effectively inhibits oxidative stress, but also alleviates inflammatory responses, mitigates endoplasmic reticulum stress, and inhibits neuronal apoptosis (Wang et al., 2022). In this study, we designed and screened a novel antioxidant, Z3, which increased the survival rate of H_2_O_2_-induced PC12 cells in a concentration-dependent manner, improved cell status, and reduced the production of MDA and ROS. Furthermore, mechanistic studies revealed that Z3 effectively activated the Nrf2/HO-1 antioxidant pathway. However, its upstream targets remain to be confirmed.

Sestrin 2 is a highly conserved oxidative stress protein that regulates cellular processes including energy metabolism, proliferation, apoptosis, and mitophagy, serving as an antioxidant in various diseases (Chen et al., 2021). Fan et al. reported that silencing of sestrin 2 exacerbates the oxidative damage induced by H_2_O_2_ in retinal ganglion cells, with mechanistic studies showing that targeting of sestrin 2 to activate the Nrf2 signaling pathway can provide protection (Fan et al., 2020). In summary, sestrin 2 effectively activates the Nrf2 antioxidant pathway to achieve cytoprotective effects. Through Western blotting experiments, we found that Z3 increased the protein expression of sestrin 2 and p62 in a concentration-dependent manner. These preliminary findings indicate that Z3 activates the sestrin 2/p62/Nrf2/HO-1 signaling pathway to exert its antioxidant activity.

Further *in vivo* experiments have demonstrated that compound Z3 significantly ameliorates the pathological progression of CIRI in MCAO-induced mice. Quantitative analysis using TTC staining of brain slices revealed that the Z3-treated group exhibited an infarct area of 11.9%, representing a 52.4% reduction compared with the NS control group (25%) and showing superior efficacy over the group treated with the clinical drug ED, which had an infarct area of 15.5%. This remarkable neuroprotective effect may stem from the unique molecular architecture of Z3, which potentially alleviates neuronal damage in the ischemic penumbra through modulation of oxidative stress cascades and suppression of mitochondrial apoptotic pathways. Given its slightly better therapeutic effect on CIRI compared with ED, we conclude that Z3 has potential for clinical development.

Although many commercial small-molecule drugs contain amide structures (Zheng et al., 2023b), the stability of these amide structures has made it difficult to further modify such drugs through condensation reactions. On the basis of the conditions explored in the early stages of this study, the synthesized diimide compounds Z1–Z5 represent a new design approach for the modification of amide structures. The lone pair of electrons on the nitrogen atom of the synthesized diimide can conjugate with two carbonyl groups, increasing the stability of the nitrogen anion form and providing a structural basis for nitrogen-containing antioxidants. Ultimately, we obtained the novel diimide antioxidant Z3 and verified its antioxidant and protective effects both *in vitro* and *in vivo*. Specifically, Z3 was found to reduce oxidative stress by activating the sestrin 2/p62/Nrf2/HO-1 signaling axis. In summary, this study has offered a new drug development strategy and a candidate drug with potential for the treatment of CIRI, as well as new ideas for functional groups with utility in the design of antioxidants.

## 5 Conclusion

In this study, five new diimide compounds (Z1–Z5) were designed and synthesized on the basis of an early-stage anti-inflammatory skeleton. Evaluation of the antioxidant activities of Z1–Z5 showed that all five compounds had significant protective effects against H_2_O_2_-induced injury in PC12 cells *in vitro*. The antioxidant activity of Z3 was the best of the five compounds, and was significantly better than that of TBHQ. A combination of network targeting and network pharmacology was employed to predict seven possible core genes of Z3-mediated treatment of CIRI. GO enrichment analysis showed that, among the top 13 biological processes of CIRI that were protected by Z3, its correlation with oxidoreductase activity was the strongest. Further investigations using MTT and colony formation assays, microscopic observation, and MDA and ROS content analyses verified that compound Z3 reduced the H_2_O_2_-induced oxidative stress of PC12 cells, thus playing an antioxidant role.

Computer molecular docking scores of Z3 with the seven core proteins predicted the strongest binding between Z3 and Nrf2; specifically, the carbon group on Z3 exhibited stable hydrogen bonding with valine 512 in Nrf2. As a key transcription factor in the Nrf2/HO-1 pathway, Nrf2 plays an important role in regulating the cellular antioxidant response involved in protecting against CIRI. Immunofluorescence, Western blotting, and ZnPP inhibitor experiments confirmed that the antioxidant mechanism of Z3 operates through Nrf2/HO-1, while TTC staining and neurobehavioral score analysis showed that Z3 had a protective effect on CIRI mice. Our findings offer a new functional group reference for the design of antioxidants, along with novel strategies for identifying targets and mechanisms of small molecule compounds, and potential drug candidates for the treatment of CIRI.

## Data Availability

The original contributions presented in the study are included in the article/Supplementary Material, further inquiries can be directed to the corresponding authors.
