# Supplementary material for: Synthesis, target analysis, and cerebroprotective effects of novel imide antioxidants via the Nrf2/HO-1 pathway in cerebral ischemia-reperfusion injury
Source: Front Pharmacol. 2025 May 2;16:1552717. doi: 10.3389/fphar.2025.1552717 (PMC12081345; doi:10.3389/fphar.2025.1552717)
Supplement: Supplementary file 1 [file DataSheet1.doc]

# Supplementary Materials

Synthesis, target analysis, and cerebroprotective effects of novel imide antioxidants via the Nrf2/HO-1 pathway in cerebral ischemia-reperfusion injury

Lili Huang ^1^, Yingqi Chen ^2^, Hua Zhou ^1^, Huihui Chen ^1^, Xiping Wu ^1^，Zhuochao Wu ^1^，Zhoudi Liu^3*^, Zhiwei Zheng ^4*^

(*1. Lihuili Hospital Affiliated to Ningbo University, Ningbo 315141, China；*

*2. Shaoxing Second Hospital, Shaoxing, 312000, China;*

*3. Department of Pharmacy, Shaoxing People’s Hospital, Shaoxing, 312035, China*

4. *Zhejiang Provincial People’s Hospital, Affiliated People’s Hospital, Hangzhou Medical College, Hangzhou, 310014, China*)

* Correspondence authors. [1083338639@qq.com](mailto:1083338639@qq.com)

Figure S1. MTT assay to detect the cytotoxicity of Z3. Cells incubated with Z3 (1.25，2.5，5 and 10 μM) for 48 h. n=3.

**H-NMR and C-NMR of compound Z1**

**H-NMR and C-NMR of compound Z2**

**H-NMR and C-NMR of compound Z3**

**H-NMR and C-NMR of compound Z4**

1. **NMR and C-NMR of compound Z5**
